# Biomimetic Porous Inorganic Materials for Bone Engineering Using a Natural Yam Stalk Template

**DOI:** 10.1021/acsomega.5c01635

**Published:** 2025-07-02

**Authors:** Bruna Borges Rossi, Elias Paiva Ferreira-Neto, Sidney José Lima Ribeiro, Gustavo Henrique de Magalhães Gomes, Clóvis Augusto Ribeiro, Diógenes Santos Dias, Isabela Louise Pereira Lopes, Érika Costa de Alvarenga, Vadim G. Kessler, Gulaim A. Seisenbaeva, Hernane Silva Barud

**Affiliations:** † Research Center on Biotechnology, University of Araraquara, Uniara, Araraquara, São Paulo 14801-309, Brazil; ‡ Institute of Chemistry, State University of São Paulo, UNESP, Araraquara, São Paulo 1480-0060, Brazil; § Department of Chemistry, Federal University of Santa Catarina, Florianopolis, Santa Catarina 88040-900, Brazil; ∥ Institute of Pure and Applied Sciences, UNIFEI, Itabira, Minas Gerais 35903-087, Brazil; ⊥ Department of Natural Science, Federal University of São João del Rei, São João del Rei, Minas Gerais 36307-352, Brazil; # Department of Molecular Sciences, BioCenter, 8095 Swedish University of Agricultural Sciences, Box 7015, Uppsala SE-75007, Sweden

## Abstract

This study explores biomimicry as a widely recognized and promising approach for developing sustainable structural materials that embody the principles of the circular economy. In this context, the study explores using yam stalks (*Dioscorea*) as a biotemplate. This natural material, composed of biopolymers such as cellulose and lignin and typically discarded as tuber waste, is characterized by a highly porous morphology with a large volume of interconnected pores. Such a structure can be used as a template to create a bone-mimicking scaffold with potential applications in tissue engineering. Through the sol–gel process and the combination of the *Dioscorea* biotemplate with tetraethyl orthosilicate (TEOS) or titanium bis­(ammonium lactate) dihydroxide (TiBALDH) precursors, silica and titania inorganic porous materials were obtained. After sol–gel deposition of inorganic oxides and removal of the *Dioscorea* biotemplate by calcination at 700 °C, scanning electron microscopy (SEM) revealed a scaffold with a homogeneous network of interconnected macropores evenly distributed throughout the material. At higher magnification, hexagonal patterns (honeycomb-like structures) were observed, highlighting the natural structural optimization that offers advantages in permeability and cellular growth. Micro CT analysis revealed total volumes of 768.61 mm^3^ for the silica-based porous scaffold and 853.00 mm^3^ for the titania-based sample, along with macropores of 203–395 and 176–286 μm per gram, respectively. This pore range is particularly suitable for cell proliferation and nutrient transport in applications like tissue engineering. Moreover, in vitro cytotoxicity and osteogenic assays showed that SD/Ti and SD/Si demonstrated promising osteogenic potential, with good cell viability, ALP activity, and collagen production in both culture media. This pore range is particularly suitable for cell proliferation and nutrient transport in applications like Tissue Engineering. Therefore, this is a promising scaffold alternative, suggesting the use of porous biomimetic materials in tissue engineering, especially synthetic bone. Furthermore, these materials offer multifunctional applications, are environmentally friendly, and are economically viable.

## Graphical abstract

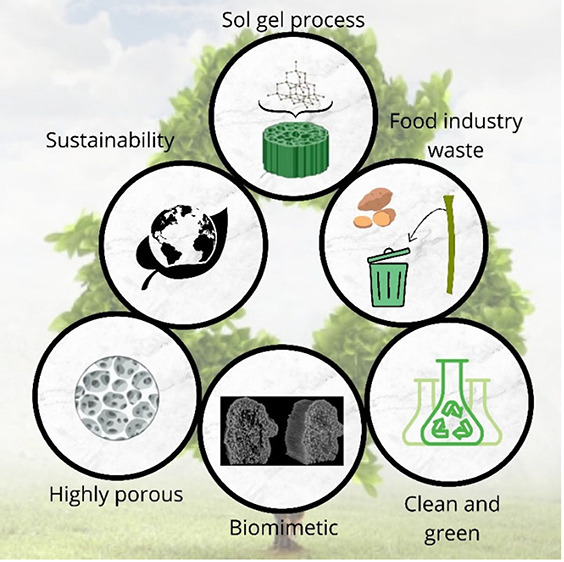

## 1 Introduction

Currently, various approaches draw inspiration from nature as a primary source of design, with biomimicry standing out as a key pathway for developing more environmentally friendly technologies. Thus, designers increasingly look to nature for inspiration and creativity in pursuing innovative and more ecologically responsible technologies. Biomimetic design (from the Greek *bios*, meaning life, and *mimesis*, meaning imitation) represents a strategy based on replicating biological structures and processes to address modern technical and environmental issues.^1^ In this context, natural structures, such as corals, marine sponges, wood, and other plant-based materials, have been explored as sacrificial templates for the fabrication of porous bioceramics that retain the morphology and multiscale porosity of the original biological material. Notably, templates of arboreal or fungal origin have been used to produce materials with macro and mesoporous architectures, showing great potential in bone tissue engineering applications.^2^ Therefore, biomimicry provides lessons on the intrinsic principles that govern perfectly designed biological systems. Biomimetic materials are thus designed to mimic and reproduce one or more characteristics of living organisms, aiming to restore natural functions or support environments by encompassing chemical, procedural, and structural aspects of materials.^3^

The technique of biomimicry can be a fundamental tool for solving current problems. Drawing on nature for creating projects, businesses, and innovative products allows challenges related to the scarcity of natural resources to be addressed and, with the aid of technology, promotes sustainability.^4^ Biomimetic materials can be employed in various fields, such as bone, nerve, and cardiovascular Tissue Engineering, as they bring principles that allow the organization of biological material structures and possess properties that mimic natural biofunctional interfaces, enabling their use in the sustainable construction of high-performance synthetic materials as potential substitutes for plastics. Plant tissues’ geometric and vascular structural similarities make them suitable for producing an inorganic matrix that may serve as structural support for cell development.^5^

In recent years, nature-inspired approaches have led to advancements in the topographic modification of biomedical materials due to promising results in in vitro studies. The diversity of natural topographies offers the potential to optimize the behavior of synthetic biomaterials in interactions with bacteria, fungi, and cells, enhancing their performance in biological environments. For instance, in the field of tissue engineering, human cells can be used to recellularize decellularized spinach leaves, revealing the potential of decellularized plants as support structures in tissue engineering. This approach may offer a “green” and cost-effective technology for large-scale regeneration of vascularized tissues.^5^ Similarly, yam stalk (*Dioscorea*), a natural material composed of cellulose and lignin derived from food industry waste, emerges as a promising biotemplate option. Due to its naturally interconnected macroporous structure, it can serve as a sacrificial template for fabricating highly porous scaffolds with potential applications in bone Tissue Engineering.

Thus, one form of biomimicry is to create scaffolds from plant tissues, in which structures provide a temporary matrix for cellular interaction and proliferation, allowing for the formation of living tissue.^6^ Plants can be readily cultivated using suitable agricultural practices in controlled environments. Yam stalk (*Dioscorea*) possesses a physical structure similar to that of human cancellous bone, making it a promising candidate for use as a porous scaffold for bone cell growth. This scaffold resembles natural bone trabeculae, displaying three-dimensional porous surfaces that mimic the extracellular matrix, thus making it suitable for supporting specific cell tissue and playing a crucial role in tissue repair and regeneration.^7,8^ This study reports a straightforward method that integrates sol–gel synthesis and biotemplating, utilizing yam stalks to fabricate silica and titania scaffolds with biomimetic porous structures. These scaffolds, derived from yam stem, composed primarily of cellulose and lignin, were characterized to evaluate their structural, morphological, and chemical properties, focusing on their potential for Tissue Engineering applications.

## 2 Experimental Section

### 2.1 Reagents

Tetraethyl orthosilicate (TEOS, Sigma-Aldrich, 98% purity) was used as the silica (SiO_2_) source. Titanium­(IV) bis­(ammonium lactate) dihydroxide (TiBALDH, 50% w/w) served as the titanium oxide (TiO_2_) source. Absolute ethyl alcohol (Neon, 99.5% P.A.) was used as the solvent. The yam stems were sourced from a plantation at the residence of one of the authors and stored in a conventional refrigerator or freezer.

### 2.2 Materials Synthesis

The process for obtaining the biocomposites involves immersing the yam stalk (*Dioscorea*) in its natural form, either prefrozen or stored in a conventional refrigerator, in the presence of TEOS/EtOH or TiBALDH/EtOH. When biopolymer-based samples are immersed in these solutions, the pores are filled with the precursor solution, and hydrolysis and condensation occur, depositing silica on the surface of the biotemplate.^9^ In the case of TiBALDH, which in reality is ammonium lactato-oxo-titanate,^10^ the addition of ethanol shifts the room-temperature equilibrium, resulting in formation of uniform 3.5 nm size lactate-capped TiO_2_ nanoparticles, which are easily adsorbed by biological surfaces.^11^ *Dioscorea* samples were cut to a thickness of approximately 0.5 cm and a diameter of 2 cm and then immersed in the solutions for 5 days. For the preparation of the TiO_2_ precursor solution, it was continuously stirred for 2 h prior to the addition of the yam stalk to ensure the formation of a homogeneous suspension. The final volume was maintained at 10 mL, with proportions of 1:1 for TEOS/ethanol and 1:4 for TiBALDH/ethanol. After immersion, the organic–inorganic material was oven-dried at 40 °C for 24h to remove ethanol, followed by thermal treatment in a muffle furnace at 700 °C for 4h to eliminate the organic component (Figure 1). The resulting porous scaffold samples after calcination were designated as SD/Si for those prepared with TEOS and SD/Ti for those prepared with TiBALDH. The as-prepared samples, following sol–gel deposition but prior to yam stalk removal, were designated as D/Si and D/Ti.

**Figure 1:**
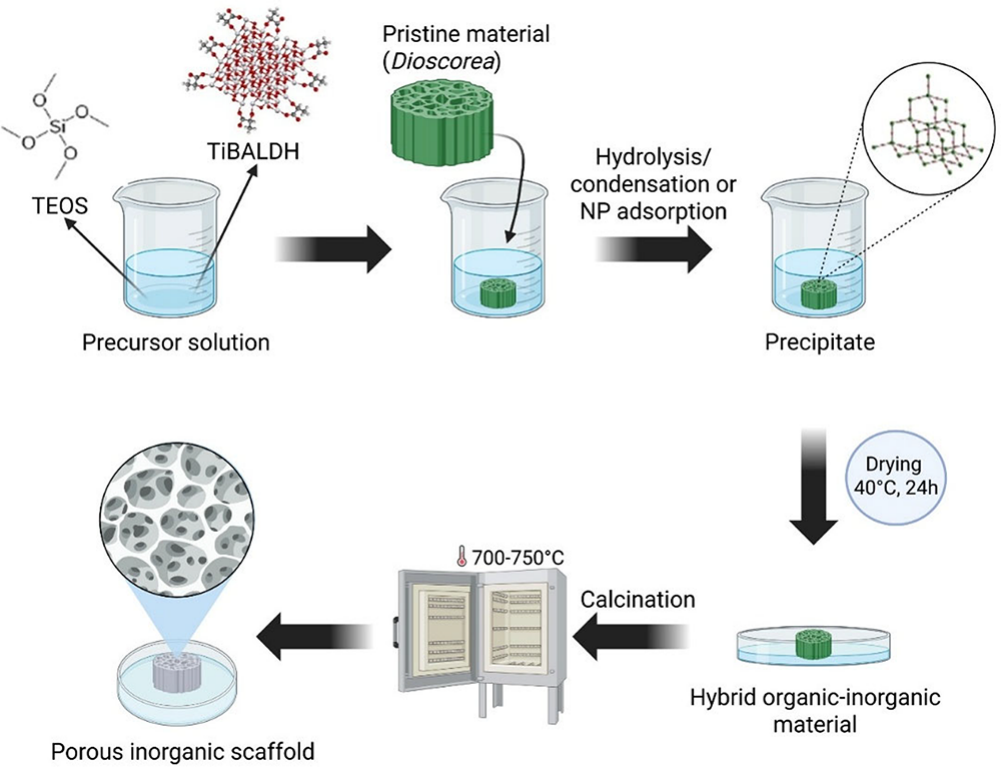
Schematic representation of the production of porous inorganic scaffolds using silica and titanium sources through the sol–gel process and *Dioscorea* (yam stalk) biotemplating.

### 2.3 Materials Characterization

The micrographs obtained by optical microscopy (OM) were performed using an optical microscope, brand Olen, model K55-TP. The OM of the SD/TiBALDH and SD/Si samples was taken at a scale of 20 μm with magnifications of 40× and 100×.

Thermogravimetric analysis (TGA-DTG) was performed to establish the thermal properties of SD/Si and SD/TiBALDH. TA Instruments equipment, using an SDT Q600 cell, was utilized. The procedure involved a temperature range from 30 to 750 °C, with a 10 °C/min rate under an oxygen gas atmosphere at a 100 mL/min flow rate. Scanning electron microscopy (SEM) analysis was conducted to evaluate the surface texture and microstructure of the SD/TiBALDH and SD/Si samples. Samples were analyzed using a field emission scanning electron microscope (FE-SEM) from JEOL, model JSM-IT500HR, equipped with secondary electron (SE) and backscattered electron (BSE) detectors. Images were captured at magnifications of 50×, 100×, and 200×. The images obtained illustrate the SD/TiBALDH and SD/Si materials at different magnifications. Pore sizes were measured using ImageJ software, and histograms for each sample were generated based on the data obtained from the software. For X-ray microtomography (μCT), samples were fixed with a standard modeling putty on a sample holder to avoid movement during the microCT run. The samples were inserted into the Skyscan 1272 CMOS Edition from Bruker Company. The run was set at 45 kV with 200 μA, an Al 0.25 mm filter, a 2048 × 2048 pixel matrix, with a 10.0 μm pixel size, a rotation step of 0.2° from 0° to 180°, frame averaging of 4, random movement of 40 pixels, and an exposure time of 658 ms per image. NRecon (Bruker) was used to reconstruct the obtained X-ray projections, using a smoothing of 1, a beam hardening correction of 5%, and no ring artifact correction. The 3D images were produced using CTVox software, which applied the correct transfer function to separate structures by low and higher density. Color-coded images of porosity and quantitative analysis were generated using CTAn software. X-ray diffraction (XRD) analysis was performed by using a D5000 X-ray diffractometer equipped with Cu Kα radiation. Measurements were carried out over a 2θ range from 4.0000° to 70.0000°, with a scan speed of 5.0000°/min and a step size of 0.0200°.

### 2.4 In Vitro Assays

#### 2.4.1 Cell Culture

The study followed the guidelines set by the National Council for Control of Animal Experimentation (CONCEA) and was approved by the UFSJ Ethics Committee for Animal Experimentation under protocol number 1096290424. Primary osteoblasts were isolated from the calvaria of neonatal Wistar rats, following the protocol previously described in earlier studies conducted by our group.^12–14^

#### 2.4.2 Viability and Cytotoxicity Tests

To assess the cytocompatibility of SD/Ti and SD/Si materials, 1 × 10^4^ cells were seeded per well in a 24-well plate onto the materials using 1 mL of DMEM high glucose culture medium (Sigma-Aldrich) supplemented with 10% fetal bovine serum (Gibco) and 1% penicillin/streptomycin (Sigma-Aldrich). After 24 h, the culture medium was replaced by medium supplied with 50 μg/mL ascorbic acid (Sigma-Aldrich) and 10 mM β-glycerophosphate (Sigma-Aldrich) in the osteogenic group (OS). After 3, 7, 10, and 14 days of incubation with the materials, cell viability was assessed using the AlamarBlue Cell Viability Reagent (Invitrogen, USA) according to the manufacturer’s instructions. Absorbance was measured at λ = 600 nm using a spectrophotometer (LMR-96-4/Loccus), and the results were expressed as a percentage of AlamarBlue reduction.^12^

#### 2.4.3 Alkaline Phosphatase (ALP) Activity

ALP activity was evaluated using the BCIP-NBT assay (5-bromo-4-chloro-3-indolyl-phosphate/nitro blue tetrazolium) (Invitrogen, USA). The supernatants from each well were removed on days 3, 7, 10, and 14, the wells were washed with PBS, and then 200 μL of BCIP-NBT solution, which was prepared according to the manufacturer’s recommended protocol, was added to each well. After 2h of incubation at 37 °C in a 5% CO_2_ atmosphere, the solution was replaced with 200 μL of SDS containing 10% of HCl, and the plates were incubated overnight at 37 °C to promote cell lysis. An optical density measurement at λ = 450 nm was performed using a spectrophotometer (LMR-96-4/Loccus).^12,15^

#### 2.4.4 Collagen Production

The supernatants from each well were collected on days 3, 7, 10, and 14 to quantify collagen production by the cells during maturation. To do this, 25 μL of the culture medium was added to 200 μL of Direct Red 80 solution (1% in saturated picric acid solution) for 1 h under gentle agitation at room temperature. The solution was centrifuged, and the pellet was washed in a 0.1 mol/L solution of acetic acid then solubilized using 150 μL of 0.1 mol/L NaOH. The optical density was measured at λ = 546 nm on a microplate spectrophotometer (LMR-96-4/Loccus).^12,13^

#### 2.4.5 Statistical Analysis

The data from the biological tests were expressed as the mean ± standard error of the mean (SEM) of *n* experiments. Differences between the groups were analyzed using Student’s *t-*test and analysis of variance (ANOVA), followed by Bonferroni’s test. A value of *P* < 0.05 was considered significant.

## 3 Results and Discussion

### 3.1 Biomimetic Inorganic Porous Scaffold Preparation and Microstructure

The sol–gel deposition of silica and titania onto the yam stalk biotemplate was evident from the whitish coloration acquired by the samples after the deposition period. Under the synthesis conditions, with a low water content (only from the 99.5% ethanol solvent), hydrolysis was facilitated by the intrinsic water content of the yam stalk. This promoted a controlled hydrolysis and condensation process at the water/solid interface, enabling the deposition of silica through a heterogeneous nucleation process, followed by the growth and aggregation of particles. The equilibrium of titania nanoparticle formation is additionally promoted by ethanol as a solvent.^10^ After sol–gel deposition, the resulting D/Si and D/Ti biocomposites were calcined to remove the organic counterpart and obtain biomimetic inorganic porous scaffolds SD/Si and SD/Ti. The high-temperature processing allows a transition from a multilayered hierarchical cell wall architecture to a support structure of pyrolyzed carbon walls, followed by the complete decomposition of organic matter. The resulting apparent physical structure of SD/Si and SD/Ti closely resembles the structure of trabecular bone, one of the main structures that make up bone tissue, along with cortical bone (compact bone), which has an internal structure that is highly porous and less dense than cortical bone.

OM (Figure 2A) micrographs provided the average pore diameters for the SD/Ti sample of approximately 40–114 and 45–35 μm for the SD/Si, which is consistent with the literature regarding scaffolds for tissue engineering, particularly for bone tissue regeneration, where pore sizes influence cell growth, vascularization, and nutrient transfer. Although the appropriate size of the mineralized pore may depend on the size of the mineralized particles and the pore sizes of similar bone tissue (50–450 μm), the range for mineralized pore size aimed at biological activity is controversial, varying between 80–250, 300–500, or 20–100 μm. Some studies identify that macropores of 20–100 μm are used for nutrition, oxygen transfer, and adsorption to enhance biomineralization activity, while pores of 100–300 μm serve as active sites rich in bone growth, vascularization, and cell proliferation.^16^

**Figure 2:**
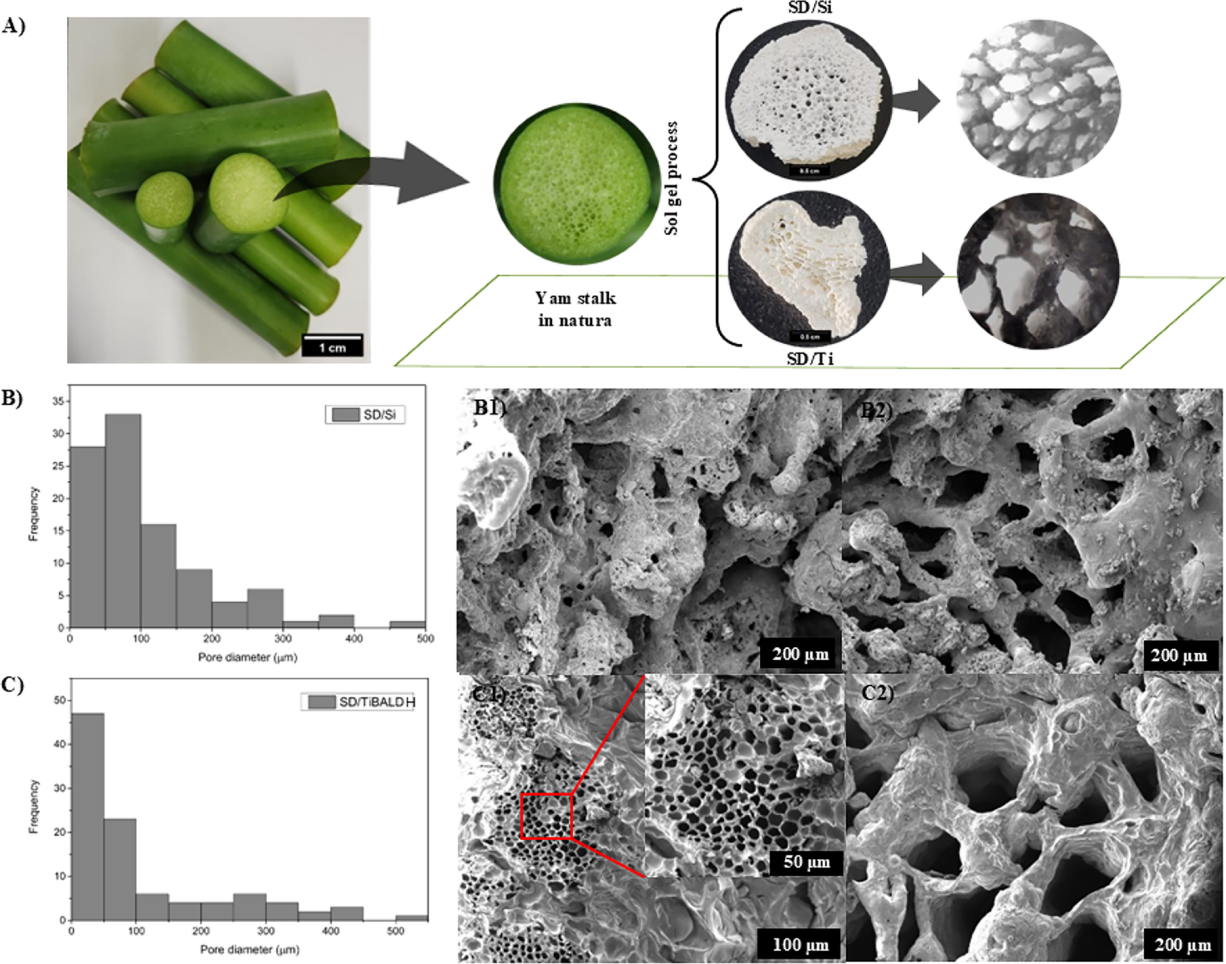
(A) Photograph of the raw yam stalk and after the sol–gel process, SD/Si and SD/Ti, along with their respective OM images. (B) Image of the histogram analysis and micrographs obtained by SEM of the SD/Si (B1 and B2) and SD/Ti (C1 and C2) samples, showing the diversity of pore sizes.

The morphology and surface of the samples were analyzed using SEM. The SEM images of SD/Ti (Figure 2C1,C2) reveal a wide range of pore sizes, from those visible to the naked eye to macropores surrounding large voids, with diameters ranging from 50 to 400 μm. The analysis at magnifications of 230× (Figure 2C1) and 55× (Figure 2C2) highlights distinct morphological features. The surface, best observed in Figure 2C2, exhibits a spicule-like texture attributed to the deposition of titania. Figure 2 displays an abundance of pores organized in a hexagonal pattern, resembling a honeycomb structure, often described in the literature as ideal for cell growth. Therefore, porous scaffolds are essential for tissue nutrition, cell proliferation, and the formation of viable new tissues. Additionally, these scaffolds also serve protective and storage functions, being used to deposit adhesion molecules and growth factors.^17^

SEM images of SD/Si (Figure 2B1,B2) exhibit a wide range of pore sizes, from those visible to the naked eye to macropores surrounding large voids with diameters ranging from 50 to 400 and 500–1000 μm. The analysis of images at 120× magnification (Figure 2B1) and 55× magnification (Figure 2B2) highlights a predominantly regular and uniform surface, albeit with roughness attributed to the coarse texture of the deposited silica material. Even regions near the material’s walls, such as those shown in Figure 2 B1, show the presence of macropores, confirming the structural heterogeneity. According to the literature, bone pores typically range from 100–300 μm, which are crucial for the proper performance of scaffolds. Criteria such as pore interconnectivity and sizes larger than 100 μm are essential for cellular growth and metabolism, allowing for vascularization and nutrient exchange. Moreover, 200–600 μm macropores, similar to those found in spongy bone, confer osteoconductive and osteointegrative properties, promoting regeneration in critical defects.^18^

### 3.2 Characterization of Molecular and Crystalline Structure (FTIR and XRD)

The FTIR spectra of the Neat Yam Stalk (NYS), SD/Si, SD/Ti, D/Si, and D/Ti samples, presented in Figures S1 (Supporting Information), reveal various characteristic bands assigned to both organic plant tissue (for samples prior to calcination) and the presence of SiO_2_ and TiO_2_ inorganic components. Specific bands and shoulders are assigned in Table S1 (Supporting Information).^19–21^ For the silica-containing samples (SD/Si and D/Si), the formation of an inorganic silica network is evidenced by the appearance of a characteristic band at 1108 cm^–1^, attributed to Si–O–Si skeletal vibrations, which overlaps with C=C and alkoxy C–O stretching modes in the uncalcined samples. Additionally, silica formation is indicated by peaks of SiO_4_ groups between 1000 and 1300 cm^–1^, the Si–OH stretching band at 960 cm^–1^ (observed only in D/Si), Si–O bending vibrations between 799 and 805 cm^–1^, and Si–O out-of-plane deformation bands between 465 and 470 cm^–1^.^22^ For the titanium-containing samples (D/Ti and SD/Ti), the broad spectral region from 1200 to 400 cm^–1^ indicates structural changes associated with the incorporation of TiO2 on the surface of the yam stalk biotemplate. The Ti–O–Ti stretching vibrations were observed between 800 and 400 cm^–1^, while Ti–O bending appeared at 670 cm^–1^, consistent with the anatase phase of TiO_2_. Moreover, the complete absence of the yam stalk characteristic vibrational bands after calcination confirms the full removal of the biotemplate.

The X-ray diffraction (XRD) results of the SD-Ti and SD-Si samples provide crucial information about each material’s crystalline structure and phase nature. For the SD-Ti sample, the observations indicate the presence of the TiO_2_ anatase phase, with characteristic peaks at 2θ of 25.4°, 37.8°, 48.1°, 54.0°, 55.0°, and 62.7°, corresponding to the crystallographic planes (101), (004), (200), (105), (211), and (204), respectively. These peaks are typical of the anatase phase of TiO_2_, a crystalline phase known for its high stability and photocatalytic properties.^23^ For the SD/Si sample, the XRD profile suggests the presence of amorphous SiO_2_, with a diffraction peak observed at 2θ of 21.2°, representing the amorphous phase of SiO_2_, which is typical of materials that do not have an ordered crystalline structure. No additional peaks corresponding to crystalline phases were observed, further reinforcing the amorphous nature of the silica present in the sample. These results indicate that while the SD/Ti sample exhibits a well-defined crystalline structure of TiO_2_ anatase, the SD/Si sample is predominantly amorphous. (Table S2 (Supporting Information)). The dynamic light scattering (DLS) analysis of TiBALDH in water (Figure S2A) and MeOH (Figure S2B) further supports these findings by showing the particle size distribution in solution, which may influence the crystallization process and the final structural properties of the samples (Figure S2 (Supporting Information)).

### 3.3 Thermogravimetric Analysis (TGA) and Derivative Thermogravimetric Analysis (DTG)

Based on the TG curves, it is possible to observe the temperature range where significant mass losses occur and the temperatures at which the maximum rate of change is observed. The TG curves of the SD/Ti and SD/Si samples reveal distinct thermal behaviors, with both samples showing an initial mass loss of up to approximately 200 °C, attributed to the removal of moisture absorbed from the environment. The principal mass loss occurs around 400 °C for SD/Ti and 550 °C for SD/Si, reflecting the maximum thermal decomposition rates. These differences in decomposition temperatures suggest that SD/Si has a slightly higher thermal stability or that interactions with silicon influence the decomposition behavior compared to titanium. After 750 °C, both samples stabilized, indicating that the main decomposition processes had been completed, with a total mass loss of approximately 0.4% for SD/Ti and 0.27% for SD/Si (Figure S3 (Supporting Information)).

### 3.4 Evaluation of Internal Pore Structure by X-ray Microtomography (μCT)

Figure 3a,c shows the X-ray transmission images of the SD/Si and SD/Ti samples, respectively, evidencing their aspect and thus confirming the biomimetic properties of the prepared materials. Interestingly, when employing the same equipment setup and conditions for both samples, the SD/Ti presented lower average grayscale values directly correlated to the material’s density, corroborating that TiO_2_ anatase has a higher density than SiO_2_. The reconstructed slices of the X-ray projections shown in Figure 3b,d resemble the internal structure of the yam stalk, presenting connected pore channels along the whole structure. This is evidence of a higher density on the external walls of the material for both samples, which is related to the anisotropic behavior of the straw-like structures, presenting a more packed region. In contrast, the center of both materials presented lower grayscale values due to thinner structures and a concentrated number of empty spaces.

**Figure 3:**
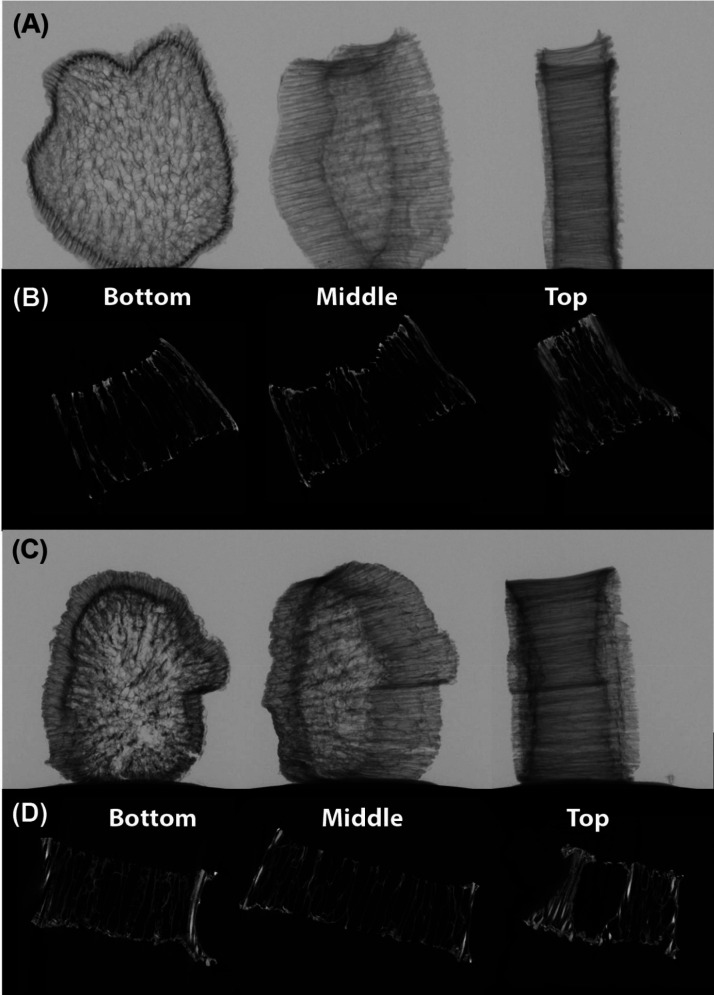
X-ray transmission image of (A) SD/Si and its respective (B) transversal reconstructed X-ray projection. Images (C, D) are regarding the SD/Ti sample.

The 3D reconstruction of SD/Si in Figure 4A corroborates the aforementioned behavior, where the outer wall of the biomimetic material presents a higher density, showing a brighter tone, which indicates a higher density. This occurs due to the anisotropic structure at the material’s outer walls, with a packed structure. The internal porous structure shows lower density and a majority concentration of pores in the material. To understand the pore distribution behavior, quantitative analysis was carried out employing the CTAn (Bruker) software, providing color-coded images of the different pore regions of the material.

**Figure 4:**
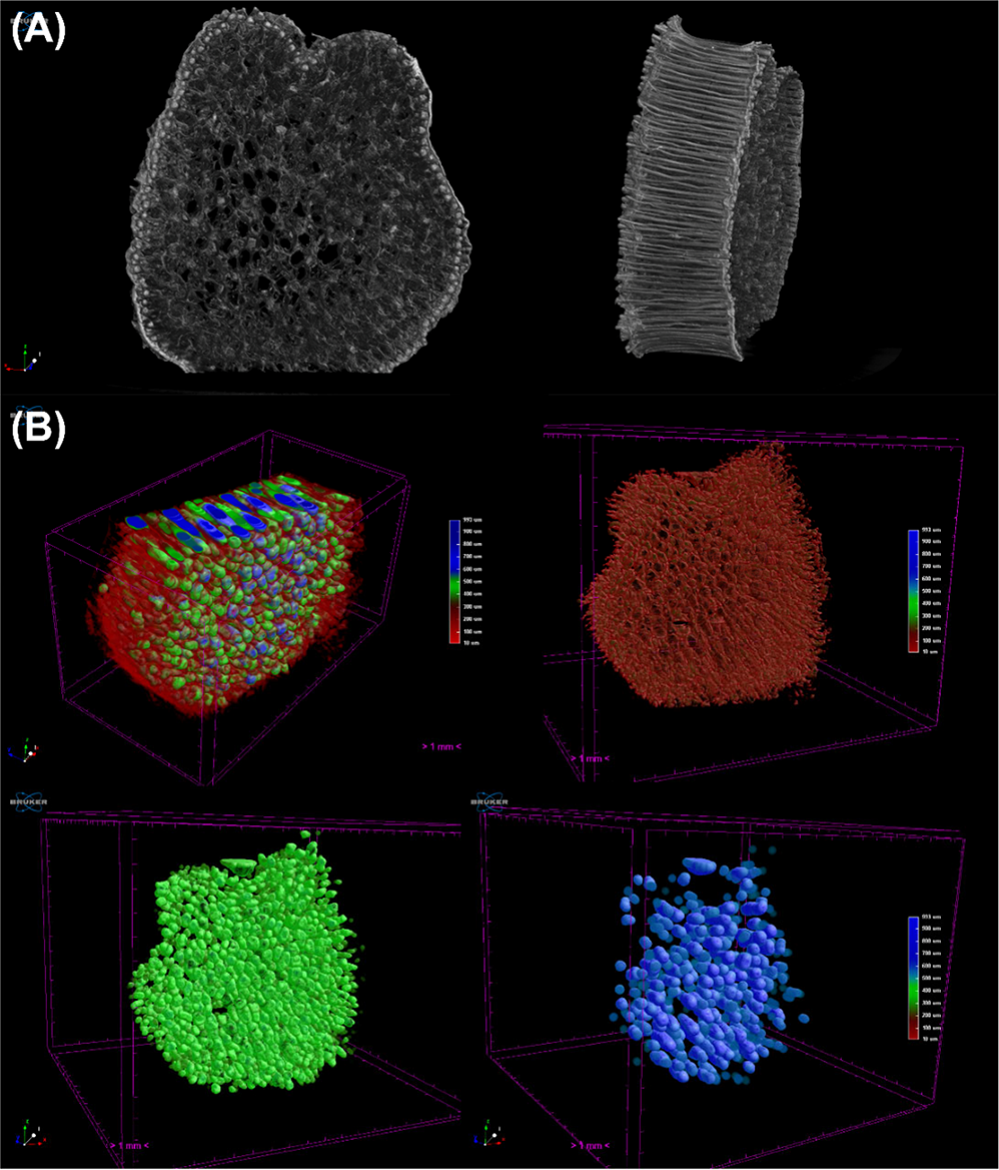
(A) 3D reconstruction of the SD/Si sample, showing its (B) color-coded images of pore size distribution, evidencing the small (red), medium (green), and large (blue) pores of the sample.

Figure 4B well illustrates this for the SD/Si, where the first image is composed of all porous structures ranging from 10 to 993 μm, with the red color regards to the small pores (10–350 μm), green the medium pores (350–550 μm), and blue the large pores (>550 μm). The composed image clearly shows the presence of medium and large pores in the center of the material; meanwhile, the red pores occur between larger pores and close to the border. This corroborates the SEM analysis, which shows a wide range of pore sizes on the structure. However, taking a look at the whole sample using the 3D model, the mimetism of bone tissue is evident, with pores ranging from 10 to 990 μm. The presence of open, tunnel-like structures in the center is clear, where the majority of these pores are represented by green and blue, where the large pores mainly occur in the center of the material.

The SD/Ti sample showed similar behavior, with slight differences in the color-coded pore size distribution. Figure 5A shows the structure, with a smaller difference in density of the outer walls and the material’s center; however, it is noticeable that the center of SD/Ti showed a more fragile structure than SD/Si, showing some faults and lack of material. Despite its behavior, the produced SD/Ti presented good biomimetic properties, with a well-defined porous structure in the center. Figure 5B shows the composed pore structure, presenting the same behavior, with tunnel-like pores in the center of the structure. These pores are mainly composed of medium and large pores; meanwhile, the red pores (smaller) occur in the whole structure. Interestingly, SD/Si was shown to be more structured than SD/Ti.

**Figure 5:**
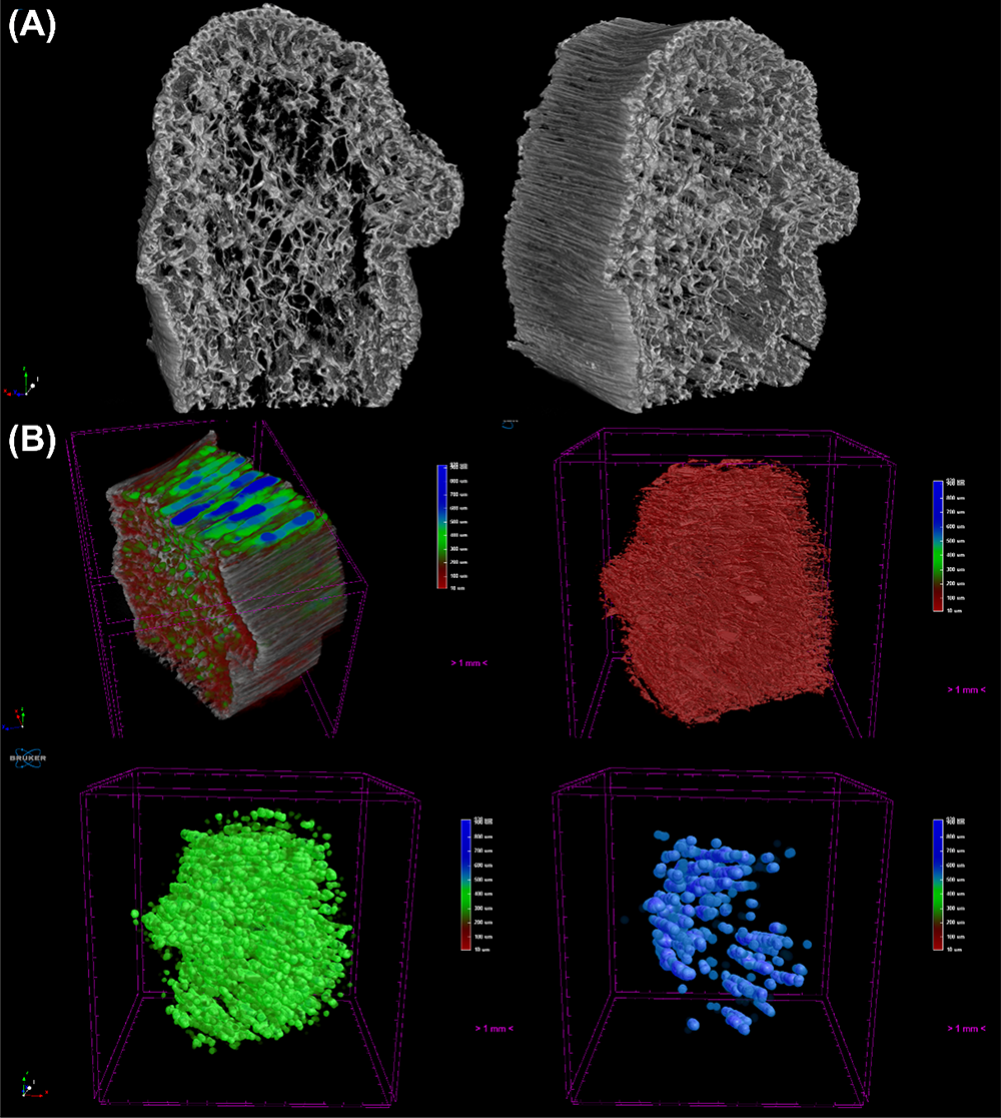
(A) 3D reconstruction of the SD/Ti sample, showing its (B) color-coded images of pore size distribution, evidencing the small (red), medium (green), and large (blue) pores of the sample.

Figure 6 shows the quantitative structure and pore size distributions for both materials. SD/Si (Figure 6a) shows smaller structures when compared with SD/Ti (Figure 6b), which is intrinsically correlated to the sol–gel synthesis procedures, where the TEOS alkoxide has a lower kinetic rate of the hydrolysis reaction, thus forming smaller structures on the yam stalk matrix. When the removal of organic content occurs, the structures are thinner. This behavior also had an impact on the pore size distribution of SD/Si (Figure 6c) and SD/Ti (Figure 6d), where the SD/Si showed a wider range of pores, centered around 400 μm and the SD/Ti around 295 μm. Despite the differences, both materials showed an adequate range of porous structures to be bone-mimicking scaffolds, with their particularities that occur due to the differences in the sol–gel chemistry of employed reactants. Table 1 shows the quantitative data obtained by pore analysis employing the CTAn software. It corroborates the aforementioned behavior, indicating that SD/Ti shows a higher mean structure size and object volume, thus leading to lower porosity and mean pore size. Despite this, SD/Ti showed a higher pore volume than SD/Si, which can be attributed to faults in the material’s center. The total porosity of both materials is around 67%, mostly composed of open pores, which is highly beneficial for the proposed applications in Tissue Engineering.

**Figure 6:**
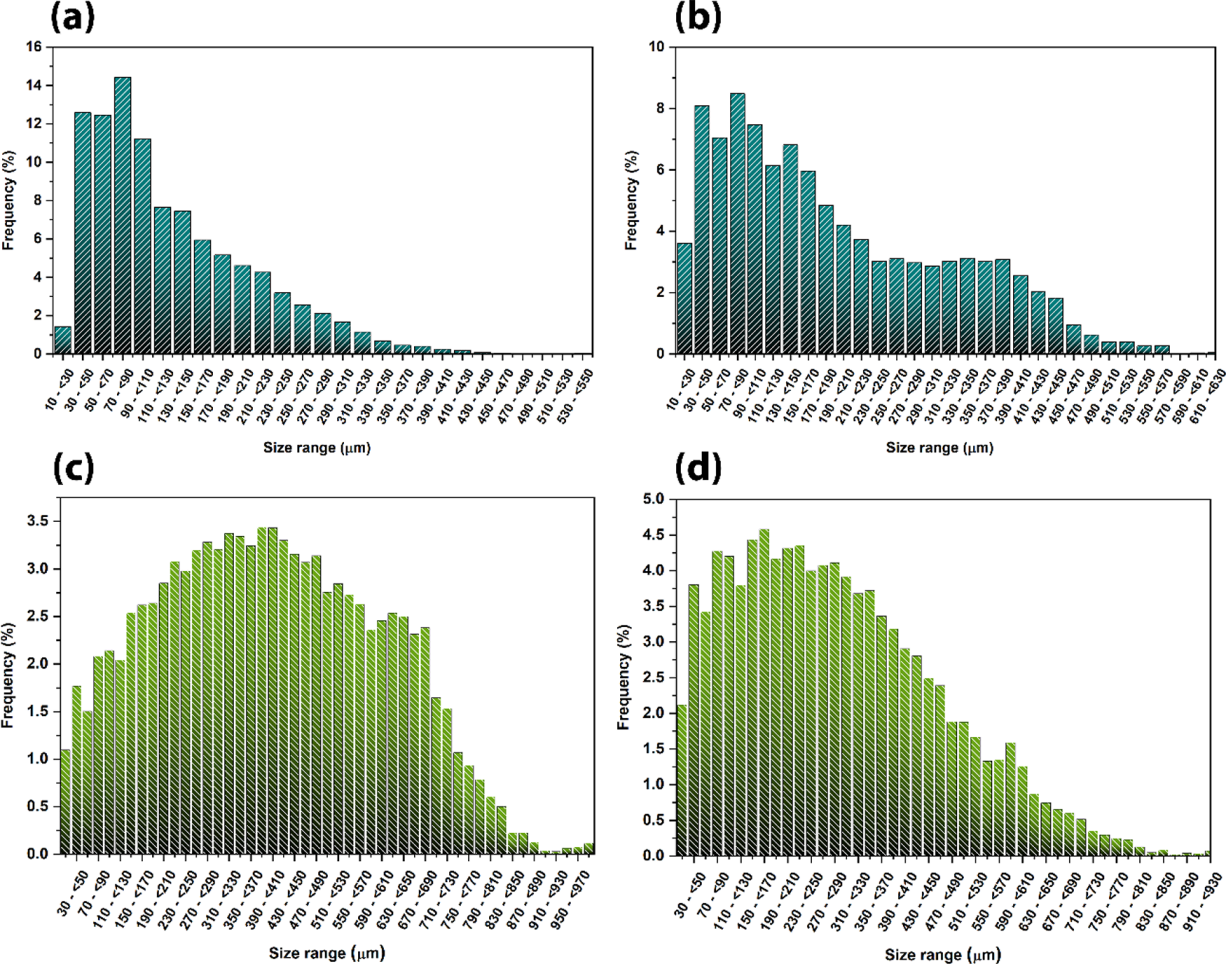
Structure thickness and pore size distribution of the (a, c) SD/Si and (b, d) SD/Ti samples, respectively.

**Table 1:** Quantitative Porosity Data of the Samples Were Obtained through MicroCT Analysis

sample	total porosity/%	open porosity/%	closed porosity/%	mean pore size/μm	mean structure size/μm	volume open pores/mm^3^	volume closed pores/mm^3^	object volume/mm^3^	total volume/mm^3^
SD/Si	67.54	67.22	0.32	395 ± 203	129 ± 81	516.71	0.82	251.08	768.61
SD/Ti	65.99	65.73	0.26	286 ± 176	191 ± 128	560.71	0.76	291.51	853.00

### 3.5 In Vitro Cytotoxicity and Osteogenic Assays

To assess the cytotoxicity and osteogenic capacity of the biomaterials, we carried out in vitro tests using primary osteoblasts under two culture conditions, basal medium, and OS medium. Our results show that in general SD/Ti and SD/Si, compared to each other, have good cell viability, ALP activity, and collagen production, regardless of the medium used (Figure 7). However, it is possible to observe significantly greater proliferation in osteoblasts cultured in basal medium at all of the times evaluated (Figure 7A), which can be explained by the stage and process of cell differentiation. OS medium induces cell differentiation, and a significant increase in ALP activity was observed, especially on days 7 and 10 for SD/Ti (OS) and SD/Si (OS) compared to SD/Ti and SD/Si cultured in basal DMEM (Figure 7B). The ALP enzyme is directly related to mineralization, since it cleaves phosphate groups in the microenvironment where it is active for the synthesis of hydroxyapatite crystals; however, ALP synthesis only begins at a more advanced stage of maturation, which consequently makes ALP activity also a marker related to osteoblast differentiation^24^ Considering, therefore, the inverse relationship normally observed between cell proliferation and differentiation in vitro assays,^25^ this result demonstrates that the reduction in proliferation observed for 7 days for SD/Ti (OS) and SD/Si (OS) can be attributed to an increase in differentiation.

**Figure 7:**
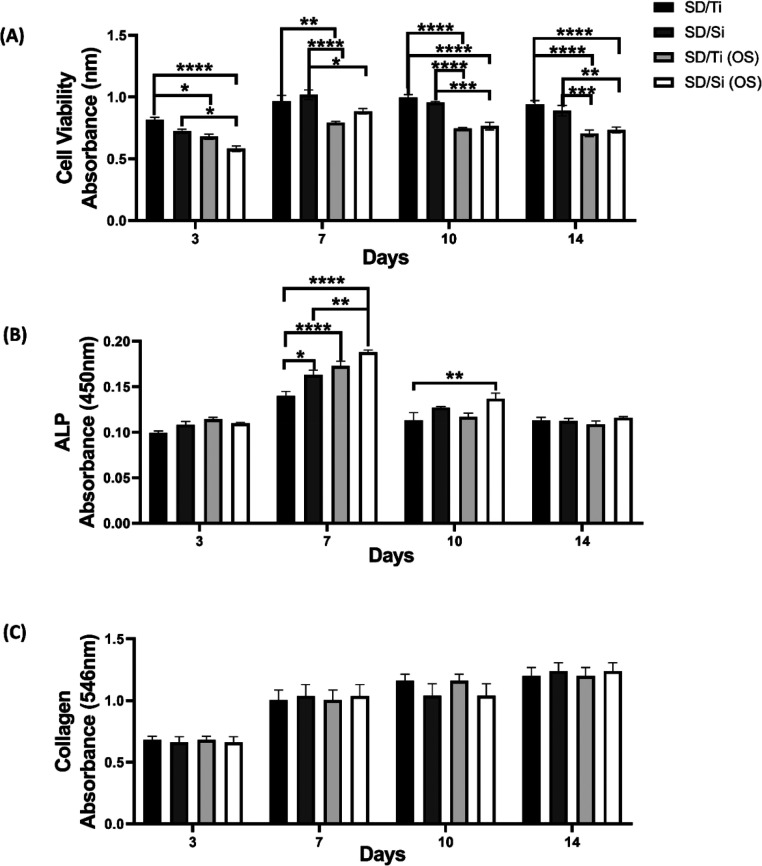
In vitro tests were performed to assess the metabolism and viability of primary osteoblasts cultured in the presence of SD/Si and SD/Ti in basal medium (DMEM) and osteogenic conditioned medium (OS). (A) Cell viability. (B) ALP activity. (C) Collagen production. Data are presented as mean ± SEM of three independent experiments (*p < 0.05, **p < 0.01, ***p < 0.001, ****p < 0.0001), analyzed using a two-way ANOVA test.

There was no significant difference in collagen production between the groups at the respective times (Figure 7C). The use of OS medium is effective in increasing osteogenic markers such as type I collagen, ALP, Runx2, and osteocalcin,^26^ so our results suggest that there was a similar increase in ALP and collagen markers for SD/Ti and SD/Si cultured in basal and OS media. Taken together, these results suggest that SD/Ti and SD/Si have promising osteogenic capacity, with similar efficacy in maintaining ALP activity and osteoblast collagen production. Nonetheless, the current results suggest that primary osteoblastic cells have the potential to form functional bone, and the ALP and collagen production assays provide a solid foundation for future investigations with this new mimetic biomaterial in bone formation in vitro and in vivo assays.

## 4 Conclusions

The results obtained demonstrate that the trabeculated yam stem, used as a three-dimensional matrix, possesses promising morphological characteristics for biomedical applications. Its topography presents a range of macropores organized in hexagonal patterns, resembling a honeycomb structure, with pore sizes compatible with spongy bone tissue. This similarity suggests significant osteoconductive and osteointegrative potential, reinforcing biomimetics as an efficient approach for the creation of silica-based inorganic scaffolds. The biomaterials SD/Ti and SD/Si demonstrated promising osteogenic potential, showing good cell viability, alkaline phosphatase (ALP) activity, and collagen production, regardless of the culture medium used (basal or OS). The basal medium supported increased cell proliferation, while the OS medium induced enhanced osteoblastic differentiation, as evidenced by a significant increase in ALP activity in the advanced stages of maturation. No significant differences in collagen production were observed between the groups, indicating similar performance. These findings suggest that SD/Ti and SD/Si effectively promote osteoblastic activity, making them potential candidates for applications in bone regeneration. Future in vitro research could focus on cell-scaffold interactions, gene expression, and in vivo assays to gain a more comprehensive understanding of these interactions. This could help optimize the design and functionality of scaffolds for tissue engineering applications. Thus, this study consolidates the use of a yam stem as a sacrificial template for the development of bioinspired materials, opening new possibilities for its application in Tissue Engineering.

## Supplementary Material



## Notes

Fourier transform infrared (FTIR) spectroscopy and characteristic bands, X-ray diffraction (XRD) patterns, dynamic light scattering (DLS) data, and thermogravimetric analysis (TGA) (PDF)
