# Supplementary material for: Biomimetic Porous Inorganic Materials for Bone Engineering Using a Natural Yam Stalk Template
Source: ACS Omega. 2025 Jul 2;10(27):29341–50. doi: 10.1021/acsomega.5c01635 (PMC12268726; doi:10.1021/acsomega.5c01635)
Supplement: Supplementary file 1 [file ao5c01635_si_001.pdf]

# Biomimetic porous inorganic materials for bone engineering using a natural yam stalk template

*Bruna Borges Rossi[1], Elias Paiva Ferreira-Neto[3], Sidney J.L. Ribeiro[2], Gustavo Henrique de Magalhães Gomes[,4], Clóvis Augusto Ribeiro[2], Diógenes Santos Dias[2]; Isabela Louise Pereira Lopes[5]; Érika Costa de Alvarenga[5]; Vadim G. Kessler[6]; Gulaim A. Seisenbaeva[6], Hernane Silva Barud [2*]*

[1]Research Center on Biotechnology – University of Araraquara-Uniara, SP, Brazil
[2] Institute of Chemistry, State University of São Paulo-UNESP, Araraquara, SP Brazil
[3] Federal University of Santa Catarina, Department of Chemistry, Florianopolis, SC Brazil
[4] Institute of pure and applied sciences – UNIFEI – Itabira, MG, Brazil
[5] Department of Natural Science, Federal University of São João del Rei - MG, Brazil.
[6] Department of Molecular Sciences, BioCenter, Swedish University of Agricultural Sciences, Box 7015, SE-75007 Uppsala, Sweden

*Corresponding authors: hsbarud@uniara.edu.br (H.S. Barud)

**Figure S1**. A) Fourier Transform Infrared (FTIR) Spectroscopy of the samples Neat Yam Stalk, D-Ti, SD-Ti (Fig. S1A) and Neat Yam Stalk, D-Si, and SD-Si (Fig. S1B). C) X-ray diffraction (XRD) of the SD-Si and SD-Ti samples.

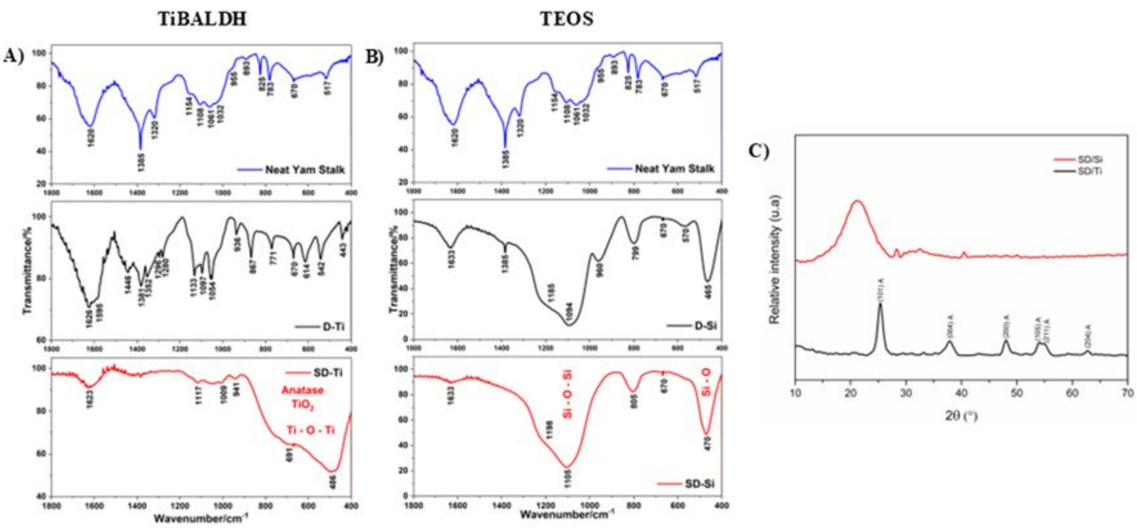

**Table S1:** Some FTIR characteristics bands for Neat Yam Stalk (NYS) Sample, D-Si and SD-Si Samples and D-Ti and SD-Ti Samples.

| **Bands /$cm^{-1}$** | **Assignments** | **Ref** |
|---|---|---|
| **Some FTIR characteristics bands for Neat Yam Stalk (NYS) Sample** | | |
| 1620 | OH bending of absorbed water; asymmetric $CO_2$ stretching for $K_2C_2O_4$ | 1 |
| 1385 | Aliphatic C-H stretching in $CH_3$ [3]; C-O stretching(w) [4]; in-the-plane CH bending [4] | 1, 3, 4 |
| 1320 | C-C and C-O ring stretching [4]; asymmetric $CO_2$ stretching for $K_2C_2O_4$ [1] | 1, 4 |
| 1154 | C-O-C ring vibrational stretching [4]; in-the-plane aromatic CH bending [14] | 1, 4 |
| 1108 | alkoxy C-O stretching [16]; ring C-C stretching [4]; Si-O stretch (Si-O-Si) [1] | 1 |
| 1061 | alkoxy C-O stretching [1]; C=C and C-C-O stretching [4]; C-O stretching (alkyl/aryl ethers) [1] | 1 |
| 1032 | Aromatic C-H in-the-plane bending plus C-O bending plus C=O stretching [3, 5] | 1,3 |
| 955 - 960 | C-H bend patterns for alkenes [1]; -HC=CH out-of-plane bending [3] | 1, 3 |
| 825, 783 | $CO_3^{2-}$ in-the-plane bending for carbonates; $MC_2O_4$ | 1 |
| 670, 570 | bending vibrations of C − H bonds in out of plane mode | 1 |
| **Some FTIR characteristics bands for D-Si and SD-Si Samples** | | |
| 1629 - 1633 | O-H stretching ; C-O bending | 2 |
| 1000 - 1300 | Si-O-Si stretching - characteristic peaks to $SiO_2$ groups | 2 |
| 960 | Si-OH stretching (*present only in D-Si sample*) | 2 |
| 799 - 805 | Si-O bending | 2 |
| 670 | Si-H wagging | 2 |
| 465-470 | Si-O out of plane deformation | 2 |
| **Some FTIR characteristics bands for D-Ti and SD-Ti Samples** | | |
| 1626 - 1595 | C=O stretching from lactate overlap with CN stretching; the absorption bands of –NH bending; Bending vibration of O-H | |

| | | |
|---|---|---|
| 1623 | Deformative vibration of Ti-OH stretching | |
| 1200 - 400 | Bands changes indicate the attachment of NYS into the composite $TiO_2$–NYS for D-Ti | |
| 800 - 400 | Ti – O – Ti stretching | |
| 670 | bending of Ti-O | |
| 486 | Ti-O bending for $TiO_2$ anatase | |

**Table S2:** The X-ray diffraction (XRD) pattern for SD/Ti and SD/Si products. XRD patterns exhibited strong diffraction peak at 25° indicating $TiO_2$ in the anatase phase (JCPDS 84-1286).

| SD/Ti – $TiO_2$ Anatase | | | SD/Si - amorphous $SiO_2$ | |
|---|---|---|---|---|
| 2θ/degree | $d_{hkl}$ (nm) | Phase | 2θ/degree | $d_{hkl}$ (nm) |
| 25.4 | 3.54 | 101 | 21.2 (max) | 4.14 (max |
| 37.8 | 2.29 | 004 | single broad peak | |
| 48.1 | 1.79 | 200 | | |
| 54.0 | 1.57 | 105 | | |
| 55.0 | 1.54 | 211 | | |
| 62.7 | 1.33 | 204 | | |
| Crystalline Index, CrI | | 32.8±0.4 % | | |
| Crystallite Size, τ | | 7.03±0.03 nm | | |

**Figure S2.** Dynamic Light Scattering (DLS) data of TiBALDH in water (A) and MeOH (B).

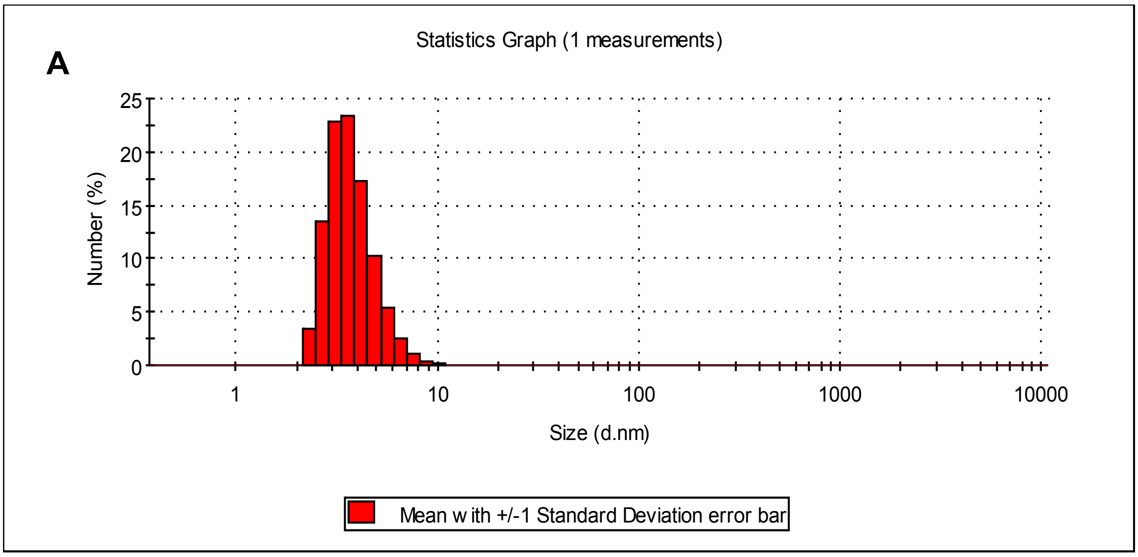


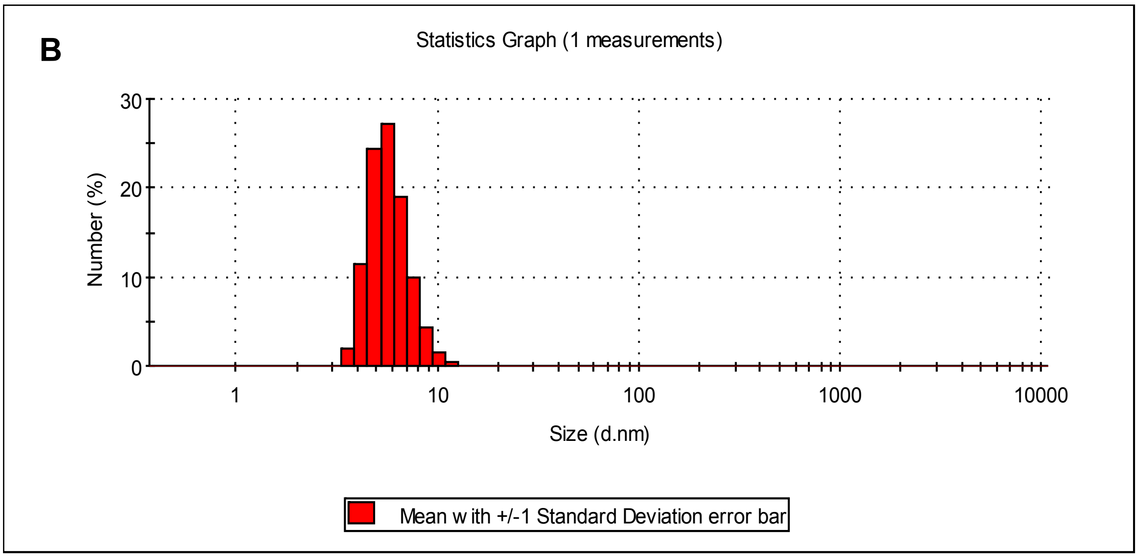

**Figure S3**. Thermogravimetric Analysis (TGA) of SD/Ti (A) and SD/Si (B) samples were conducted from 0 to 750 °C in an oxygen atmosphere.

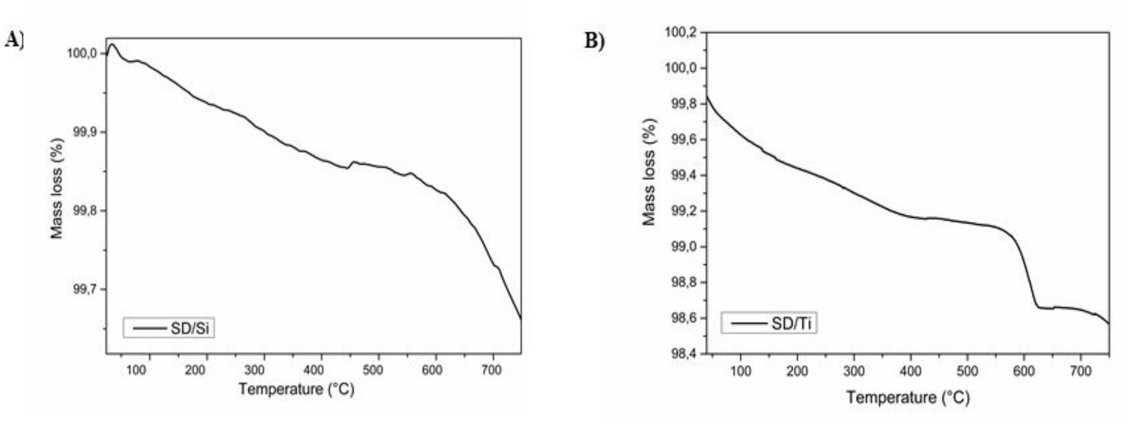


**References:**


1. Dias, D.S., Faria, F.A., Mattiolli, L. et al. Moisture sorption of biochar from banana pseudostem fibers according to the pyrolysis temperature. J Therm Anal Calorim 138, 3825–3832 (2019). https://doi.org/10.1007/s10973-019-08141-8
2. Ref: Journal of Optoelectronics and Advanced Materials Vol. 7, No. 1, February 2005, p. 389 – 392.
3. Faix O. Classication of Lignins from Dierent Botanical Origins by FT-IR Spectroscopy. Holzforschung, 1991; 45(s1):21-28.
4. Adapa PK, Karunakaran C, Tabil LG, Schoenau GJ. 2009. Potential Applications of Infrared and Raman Spectromicroscopy for Agricultural Biomass. Agricultural Engineering International: the CIGR Ejournal, Manuscript 1081Vol. XI(February): 1-25.
5. Pandey KK. A study of chemical structure of soft and hardwood and wood polymers by FTIR spectroscopy. Journal of Applied Polymer Science 1999; 71(12):1969-1975
